# Evaluation of computer-based computer tomography stratification against outcome models in connective tissue disease-related interstitial lung disease: a patient outcome study

**DOI:** 10.1186/s12916-016-0739-7

**Published:** 2016-11-23

**Authors:** Joseph Jacob, Brian J. Bartholmai, Srinivasan Rajagopalan, Anne Laure Brun, Ryoko Egashira, Ronald Karwoski, Maria Kokosi, Athol U. Wells, David M. Hansell

**Affiliations:** 1 Department of Radiology, Royal Brompton Hospital, Royal Brompton and Harefield NHS Foundation Trust, London, SW3 6NP UK; 2 Division of Radiology, Mayo Clinic Rochester, Rochester, Minnesota USA; 3 Department of Physiology and Biomedical Engineering, Mayo Clinic Rochester, Rochester, Minnesota USA; 4 Interstitial Lung Disease Unit, Royal Brompton Hospital, Royal Brompton and Harefield NHS Foundation Trust, London, UK; 5 Department of Radiology, Royal Brompton Hospital, Royal Brompton and Harefield NHS Foundation Trust, London, SW3 6NP UK

**Keywords:** Connective tissue disease, Computer tomography, Quantitative CT, Interstitial lung disease, Pulmonary fibrosis

## Abstract

**Background:**

To evaluate computer-based computer tomography (CT) analysis (CALIPER) against visual CT scoring and pulmonary function tests (PFTs) when predicting mortality in patients with connective tissue disease-related interstitial lung disease (CTD-ILD). To identify outcome differences between distinct CTD-ILD groups derived following automated stratification of CALIPER variables.

**Methods:**

A total of 203 consecutive patients with assorted CTD-ILDs had CT parenchymal patterns evaluated by CALIPER and visual CT scoring: honeycombing, reticular pattern, ground glass opacities, pulmonary vessel volume, emphysema, and traction bronchiectasis. CT scores were evaluated against pulmonary function tests: forced vital capacity, diffusing capacity for carbon monoxide, carbon monoxide transfer coefficient, and composite physiologic index for mortality analysis. Automated stratification of CALIPER-CT variables was evaluated in place of and alongside forced vital capacity and diffusing capacity for carbon monoxide in the ILD gender, age physiology (ILD-GAP) model using receiver operating characteristic curve analysis.

**Results:**

Cox regression analyses identified four independent predictors of mortality: patient age (*P* < 0.0001), smoking history (*P* = 0.0003), carbon monoxide transfer coefficient (*P* = 0.003), and pulmonary vessel volume (*P* < 0.0001). Automated stratification of CALIPER variables identified three morphologically distinct groups which were stronger predictors of mortality than all CT and functional indices. The Stratified-CT model substituted automated stratified groups for functional indices in the ILD-GAP model and maintained model strength (area under curve (AUC) = 0.74, *P* < 0.0001), ILD-GAP (AUC = 0.72, *P* < 0.0001). Combining automated stratified groups with the ILD-GAP model (stratified CT-GAP model) strengthened predictions of 1- and 2-year mortality: ILD-GAP (AUC = 0.87 and 0.86, respectively); stratified CT-GAP (AUC = 0.89 and 0.88, respectively).

**Conclusions:**

CALIPER-derived pulmonary vessel volume is an independent predictor of mortality across all CTD-ILD patients. Furthermore, automated stratification of CALIPER CT variables represents a novel method of prognostication at least as robust as PFTs in CTD-ILD patients.

**Electronic supplementary material:**

The online version of this article (doi:10.1186/s12916-016-0739-7) contains supplementary material, which is available to authorized users.

## Background

Computed tomography (CT) evaluation of patients with individual connective tissue disease-related interstitial lung diseases (CTD-ILDs) have shown that several parenchymal patterns, including honeycombing [1, 2], reticulation [3], and fibrosis extent, are associated with a poor outcome [1, 4–6]. However, while studies of prognostic indices within individual CTDs convey valuable information about specific, small patient groups, the applicability of such indices to a wider group of “all-comers” CTDs needs validation.

The importance of identifying prognostic indices across a population of various CTD diagnoses lies in the fact that CTD sub-groups often overlap both in their clinical and CT characteristics. Yet, there are very few CT studies that have considered mixed populations of CTD patients. One such study, by Walsh et al. [7], identified severity of traction bronchiectasis and honeycombing as indices predictive of mortality, confirming the importance of two parenchymal patterns previously shown to be prognostically important in the non-CTD idiopathic interstitial pneumonias [8–10].

Computer-based CT analysis in the CTDs [11, 12] has been relatively neglected when compared to idiopathic pulmonary fibrosis (IPF) [13–15]. Furthermore, the application of advanced mathematical modelling techniques to CT datasets has been limited thus far [16] despite the modelling of quantified CT variables having the potential to provide a comprehensive morphological analysis of a patient’s disease. By evaluating the entirety of a CT dataset, computer tools, when allied to modelling techniques, can identify patient clusters that share similar disease phenotypes and potentially identify sub-groups with similar outcomes.

The current study therefore compared the strength of visual and computer-based (CALIPER) CT patterns and pulmonary function tests (PFTs) for the prediction of mortality for a mixed cohort of CTD-ILD patients. A secondary analysis evaluated mortality prediction across the entire cohort using mathematical modelling of CALIPER-scored CT variables and compared mortality prediction against the interstitial lung disease gender, age physiology (ILD-GAP) outcome model.

## Methods

### Study cohort

A retrospective analysis of an ILD database identified all new consecutive patients with a multidisciplinary diagnosis of CTD-ILD, diagnosed according to published guidelines [17] over a 4.5-year period (January 2007 to July 2011). Underlying CTD diagnoses were defined according to the relevant rheumatology diagnostic guidelines [18–24]. Patients with a non-contrast, supine, volumetric thin section CT were captured, and subsequent exclusions are shown as per the CONSORT diagram in Additional file 1: Figure S1. Approval for this analysis of clinically indicated CT and pulmonary function data was obtained (and patient consent was waived) from the Institutional Ethics Committee of the Royal Brompton Hospital and the Institutional Review Board of the Mayo Clinic.

### Study protocols

CT, CALIPER and PFT protocols have been previously described [25]. PFTs analysed included forced expiratory volume in one second (FEV1), forced vital capacity (FVC), total lung capacity (TLC), transfer coefficient of the lung for carbon monoxide (Kco), single breath carbon monoxide diffusing capacity corrected for haemoglobin concentration (DLco), and the composite physiologic index (CPI) [26].

### CT evaluation

**Fig. 1:**
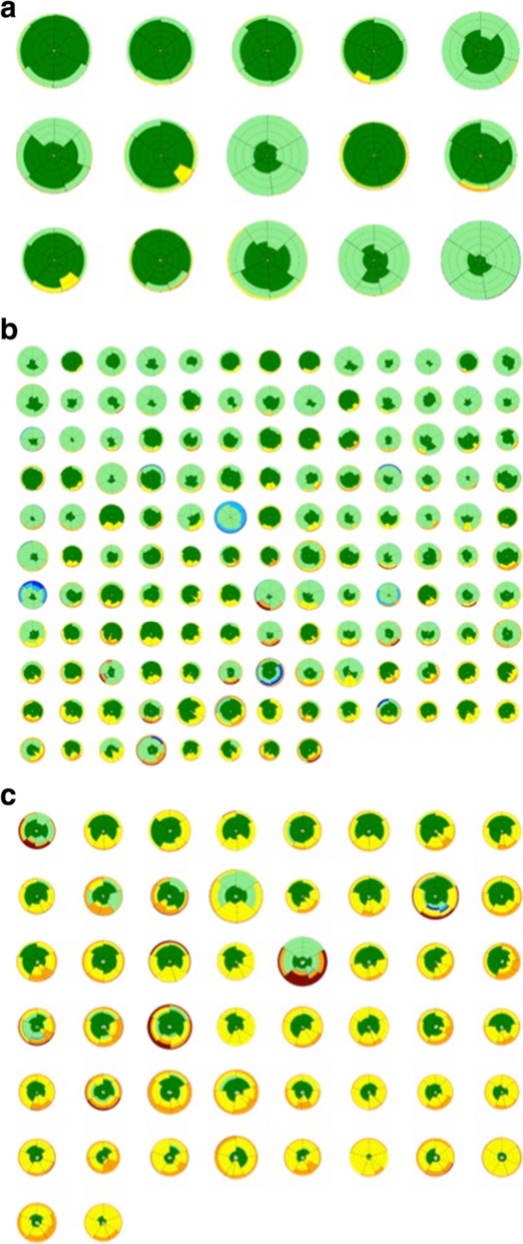
**a**–**c** Glyphs demonstrating the CT parenchymal pattern extents of each patient in each of the three connective tissue disease-related interstitial lung disease groups (**a** = Group 1 (n = 15); **b** = group 2 (n = 138); **c** = group 3 (n = 50)) derived following CALIPER CT analysis. Each glyph comprises six wedges, corresponding to lung zones (upper, middle and lower for each lung). The size of a wedge reflected the volume of the zone relative to the total lung volume. Within each lung zone, every voxel was classified into one of eight separately colour coded CALIPER parenchymal patterns: ground glass opacity, yellow; reticular pattern, orange; honeycombing, brown; Grade 1 decreased attenuation (DA), light green; Grade 2 DA, light blue; Grade 3 DA, dark blue; Normal lung, dark green; pulmonary vessel volume (PVV; pulmonary arteries and veins, excluding vessels at the lung hilum), white. The relative volumes of the patterns within a zone determined the proportions of each colour in a zone; dotted concentric lines represent quintiles of lung volume

Each CT scan was evaluated independently by two radiologists (AB, RE) with 7 and 9 years thoracic imaging experience, respectively, blinded to all clinical information [25]. Visual CT parameters included ground glass opacity, reticular pattern, honeycombing, emphysema, consolidation, mosaicism (decreased attenuation component), and traction bronchiectasis as described in Additional file 1: Appendix. CALIPER evaluation of the lungs [13] is described in Additional file 1: Appendix and was pictorially expressed as a glyph [27] (Fig. 1). Total fibrosis extent represented the sum of reticulation and honeycombing, whilst total ILD extent additionally summed ground glass opacification. All CT variables were expressed as a percentage, to the nearest 5%, of the total lung volume except traction bronchiectasis which was scored using a categorical 4-point lobar scale [25].

### Stratification of CALIPER-derived parenchymal pattern extents

Within each of three lung zones (upper, middle and lower), CALIPER evaluated parenchymal pattern extents in both the medial and lateral regions of a zone [13]. Within the resulting 12 zones, global and regional dissimilarities in the eight CALIPER-quantified patterns (ground glass opacity, reticular pattern, honeycombing, grade 1 low attenuation areas (LAA), grade 2 LAA, grade 3 LAA, and normal lung and pulmonary vessel volume (PVV)) were evaluated by a dissimilarity metric [16]. The dissimilarity metric evaluated regional dissimilarities in lung volume separately within each lung as a proportion of the total lung volume. Between two individual lungs, dissimilarities in the proportions of absolute lung volumes in corresponding regions as well as dissimilarities in the proportions of specific parenchymal patterns in the corresponding regions were also calculated.

The dissimilarity metric was used to compare all 203 CTD-ILD cases in a pairwise manner. The resultant 203 × 203 matrix was stratified using single pass unsupervised affinity propagation [28] to identify unique clusters that represented patient groups with common parenchymal features. No pre-test designation as to the number of expected clusters was necessary, as affinity propagation derives naturally occurring clusters using real-valued message exchange [28].

### Statistical analysis

Data are given as means with standard deviations, or numbers of patients with percentages where appropriate. Interobserver variation for visual scores was assessed using the single determination standard deviation. Linear and logistic regression analyses were used to examine relationships between PVV and CT, echocardiographic and functional variables. Univariate and multivariate Cox proportional hazards analyses were used to investigate relationships within and between the three data sets: CALIPER CT evaluation, visual CT evaluation and PFTs. Variables were removed from multivariate models in a stepwise manner at a 0.01 level of significance.

Differences in functional and morphological indices between groups created following automated stratification of CALIPER parenchymal pattern scores were examined using one-way analysis of variance (ANOVA) and post-ANOVA pairwise t-test analyses with the Bonferroni correction applied for multiple analyses. Cox regression analysis and Kaplan–Meier survival curves compared using the Log rank test were used to identify survival differences between automated stratified groups.

### Analyses using patient outcome models

The ILD-GAP model, a staging system determining patient outcome, was evaluated in the current study against the automated stratified CTD-ILD groups. The ILD-GAP model categorically weighs four variables (age, gender, FVC and DLco) and generates a 4-point categorical scale from an 8-point score [29].

In the primary analysis between outcome models, the ability of automated stratified CALIPER-CT groups to substitute for the pulmonary function variables (FVC and DLco) in the ILD-GAP model was investigated. The automated stratified groups were converted into a 5-point categorical scale in line with the 5-point weighting of FVC and DLco in the ILD-GAP score, from which the ILD-GAP model is derived. Stratified group 1 patients were assigned a score of 0, stratified group 2 patients a score of 2, and stratified group 3 patients a score of 4. Gender and age were scored on 2- and 3-point scales in accordance with the ILD-GAP score and were combined with the stratified group scores to create an 8-point scale (“Stratified-CT score”). The reason for the 5-point weighting of the automated stratified groups was to maintain the weighting of age and gender in the Stratified-CT score when compared to the ILD-GAP model, where the weighting of FVC (0,1,2) and DLco (0,1,2) was spread across a 5-point scale. Had a 3-point scale been used for the automated stratified groups, in the subsequently created models, patient age and gender would have been as powerful in determining outcome as the CT variables (stratified groups), which would have biased our results when comparisons to the ILD-GAP index were evaluated.

The 8-point Stratified-CT score was condensed into a 4-point model in line with the ILD-GAP model and was termed the “Stratified-CT model”, where a score of 0/1 represented grade 1, a score of 2/3 represented grade 2, a score of 4/5 represented grade 3, and a score over 5 represented grade 4. Finally, the automated stratified groups (measured on a 3-point scale) were combined with the ILD-GAP model (which amalgamated patient age, gender, FVC and DLco in an 8-point ILD-GAP score as previously described and was then converted into a 4-point ILD-GAP model) to form a “Stratified CT-GAP model”.

The predictive power of the Stratified CT model, the ILD-GAP model and the Stratified CT-GAP model to determine mortality in the same 179 patients was evaluated using univariate and multivariate Cox mortality analyses with bootstrapping of 1000 randomly generated samples as well as receiver operator characteristic (ROC) curve analysis. Statistical analyses were performed with IBM SPSS Statistics for Macintosh, Version 20.0. Armonk, NY: IBM Corp.

## Results

### Cohort analysis

A total of 203 patients were identified with the following CTD diagnoses: rheumatoid arthritis (RA, n = 50), systemic sclerosis (n = 65), overlap CTD (n = 36, polymyositis and dermatomyositis (n = 23), mixed connective tissue disease (n = 16), primary Sjögren’s syndrome (n = 10), and systemic lupus erythematosus (SLE, n = 3); 69% of the CTD cohort were female, 60% had never smoked, and 65% were still alive after a mean follow-up time of 46 months.

### Baseline CT analysis

Visual scoring generally identified more extensive ILD and emphysema than CALIPER across all groups (Table 1). ILD was mainly comprised of ground glass opacity on CALIPER but consisted of slightly more extensive reticular pattern than ground glass opacity on visual scoring. Interobserver agreement between the visual scorers is provided in Additional file 1: Table S2. Differences in disease extents between ILD-GAP groups are shown in Additional file 1: Table S3.

**Table 1:** Patient age, gender, smoking status and measures of pulmonary function indices, CALIPER and visually scored CT parameters and echocardiography data in patients with connective tissue disease-related interstitial lung disease with a subanalysis for each of three separate groups derived following mathematical modelling using automated stratification

Variable	All CTD	Stratified Group 1	Stratified Group 2	Stratified Group 3
Units are percentage unless stated	(n = 203 unless stated)	(n = 15 unless stated)	(n = 138 unless stated)	(n = 50 unless stated)
Median age, years	58	58	59	53
Male/female	63/140	2/13	47/91	14/36
Survival (alive/dead)	131/72	14/1	95/43	22/28
Never/ex-smokers	122/76	9/6	83/52	30/18
Follow-up time, months	45.9 ± 24.0	57.8 ± 15.3	47.4 ± 22.2	38.1 ± 28.5
FEV1 % predicted	68.7 ± 18.4 (184)	79.5 ± 14.7 (14)	71.4 ± 18.3 (122)	58.7 ± 15.4 (48)
FVC % predicted	70.8 ± 20.9 (184)	88.3 ± 15.6 (14)	74.4 ± 20.7 (122)	56.7 ± 13.9 (48)
DLco % predicted	39.3 ± 14.2 (189)	54.5 ± 14.4 (14)	41.6 ± 13.0 (129)	28.4 ± 9.8 (46)
Kco % predicted	66.1 ± 17.6 (189)	72.2 ± 17.4 (14)	68.0 ± 16.8 (129)	58.9 ± 18.0 (46)
TLC % predicted	70.9 ± 16.7 (175)	89.6 ± 13.6 (13)	72.7 ± 16.0 (121)	59.4 ± 11.2 (41)
CPI	51.2 ± 13.7 (180)	35.8 ± 11.9 (14)	48.6 ± 12.6 (120)	62.7 ± 8.0 (46)
CALIPER ILD extent	20.2 ± 18.3	2.4 ± 2.1	12.7 ± 8.7	46.2 ± 15.0
CALIPER Fibrosis extent	5.5 ± 5.2	0.7 ± 0.5	4.0 ± 3.4	11.0 ± 5.9
CALIPER GGO	14.7 ± 15.7	1.7 ± 1.9	8.7 ± 7.8	35.2 ± 16.3
CALIPER Reticular pattern	4.9 ± 4.6	0.6 ± 0.4	3.7 ± 3.0	9.8 ± 5.3
CALIPER Honeycombing	0.6 ± 1.5	0.1 ± 0.1	0.4 ± 0.7	1.2 ± 2.8
CALIPER Emphysema	0.6 ± 2.8	0.1 ± 0.1	0.9 ± 3.3	0.2 ± 0.7
CALIPER PVV	4.2 ± 1.7	2.5 ± 0.5	3.6 ± 1.1	6.5 ± 1.3
CALIPER Normal lung	74.9 ± 19.5	95.1 ± 2.3	82.8 ± 9.5	47.1 ± 15.2
Visual ILD extent	53.5 ± 24.8	43.1 ± 28.4	47.4 ± 23.0	73.5 ± 17.0
Visual fibrosis extent	31.7 ± 19.9	7.2 ± 6.9	27.6 ± 16.4	50.6 ± 16.7
Visual GGO	20.7 ± 20.0	34.1 ± 28.8	18.9 ± 19.1	21.7 ± 17.9
Visual reticular pattern	27.6 ± 17.9	7.2 ± 6.9	24.9 ± 15.4	41.1 ± 17.7
Visual honeycombing	4.1 ± 9.3	0.0 ± 0.0	2.7 ± 6.6	9.4 ± 14.1
Visual consolidation	0.6 ± 2.3	1.8 ± 4.9	0.6 ± 2.2	0.4 ± 1.0
Visual emphysema	4.1 ± 10.7	5.0 ± 16.9	3.8 ± 9.8	4.8 ± 10.9
Visual mosaicism	1.7 ± 4.9	3.9 ± 8.1	1.4 ± 4.9	1.9 ± 3.6
Visual TxBx (max score 18)	5.5 ± 3.7	1.0 ± 1.2	5.1 ± 3.4	7.9 ± 3.6
Main PA diameter, mm	31.0 ± 4.8	28.7 ± 4.0	30.4 ± 4.3	33.3 ± 5.6
AAo diameter, mm	32.0 ± 4.0	31.9 ± 3.3	31.8 ± 4.0	32.7 ± 4.0
RVSP, mmHg	39.2 ± 16.1 (100)	28.9 ± 3.8 (7)	37.0 ± 14.7 (62)	45.8 ± 18.1 (31)

Data represent mean values with standard deviations. *CTD* connective tissue disease, *FEV1* forced expiratory volume in one second, *FVC* forced vital capacity, *DLco* diffusing capacity for carbon monoxide, *Kco* carbon monoxide transfer coefficient, *TLC* total lung capacity, *CPI* composite physiologic index, *ILD* interstitial lung disease, *GGO* ground glass opacity, *PVV* pulmonary vessel volume, *TxBx* traction bronchiectasis, *PA* pulmonary artery, *AAo* ascending aorta, *RVSP* right ventricular systolic pressure

To further evaluate the PVV variable, relationships with markers of interstitial disease and pulmonary vascular disease were explored. On linear regression analyses, PVV demonstrated strong linkages with CALIPER ILD extent (R^2^ = 0.73, *P* < 0.0001) and visual ILD extent (R^2^ = 0.39, *P* < 0.0001) but only weak associations with RVSP (R^2^ = 0.09, *P* = 0.002) and Kco (R^2^ = 0.05, *P* = 0.002).

### Mortality analysis

On univariate mortality analysis, predictors of mortality included CALIPER and visual measures of fibrosis including reticular pattern, honeycombing, and ILD and fibrosis extents as well as visual traction bronchiectasis and CALIPER PVV (Table 2). Of the pulmonary function indices, DLco, Kco, and the CPI were strong univariate predictors of mortality (Table 2). Patient age and a positive smoking history were also strongly linked to mortality. Univariate mortality analyses were also performed for the continuous scores (prior to their categorization into indices) of the three models: ILD-GAP, Stratified CT, and Stratified CT-GAP models (Table 2).

**Table 2:** Univariate Cox regression analysis of connective tissue disease-related interstitial lung disease (CTD-ILD) cases demonstrating variables significantly predictive of mortality: CALIPER indices (top white), visually derived high-resolution computed tomography indices (light grey) other indices (dark grey)

	Number of patients	Hazard ratio	*P* value	95% Confidence interval	
				Lower	Upper
CALIPER score					
Total ILD extent	203	1.02	0.002	1.01	1.03
Total fibrosis extent	203	1.12	<0.0001	1.08	1.16
Reticular pattern	203	1.12	<0.0001	1.08	1.16
Honeycombing	203	1.18	0.0004	1.08	1.29
Emphysema	203	1.08	0.004	1.02	1.13
Pulmonary vessel volume	203	1.37	<0.0001	1.19	1.57
VISUAL score					
ILD extent	203	1.02	0.001	1.01	1.03
Fibrosis extent	203	1.03	<0.0001	1.02	1.04
Reticular pattern	203	1.02	0.002	1.01	1.03
Honeycombing	203	1.03	<0.0001	1.02	1.05
Traction bronchiectasis	203	1.16	<0.0001	1.09	1.24
Pulmonary artery diameter	203	1.09	0.0002	1.04	1.15
DLco	189	0.96	0.0004	0.94	0.98
Kco	189	0.97	0.0003	0.96	0.99
CPI	180	1.04	0.002	1.01	1.06
Age	203	1.06	<0.0001	1.04	1.09
Previous or current smoker	203	2.21	0.001	1.40	3.61
ECHOCARDIOGRAPHY RVSP	100	1.03	<0.0001	1.02	1.05
Continuous Model Score					
ILD-GAP score	179	1.74	<0.0001	1.45	2.08
Stratified CT score	179	2.00	<0.0001	1.63	2.45
Stratified CT-GAP	179	1.65	<0.0001	1.41	1.94
MULTIVARIATE MODEL					
Age		1.07	<0.0001	1.04	1.10
Previous/current smoker		2.87	0.0003	1.63	5.04
Pulmonary vessel volume		1.57	<0.0001	1.35	1.82
Kco		0.98	0.003	0.96	0.99

A multivariate model demonstrating independent predictors of mortality is also shown (bottom white).

*ILD* interstitial lung disease, *FVC* forced vital capacity, *DLco* diffusing capacity for carbon monoxide, *Kco* carbon monoxide transfer coefficient, *CPI* composite physiologic index, *RVSP* right ventricular systolic pressure

A combined multivariate analysis of the CTD cohort included CALIPER and visual CT variables, pulmonary function indices, and patient age and smoking history (Table 2). DLco, Kco and CPI were each inserted into the model as they demonstrated similar significance with regard to mortality on univariate analysis. In the combined model, patient age, smoking history, Kco and PVV were the four variables independently predictive of mortality (Table 2). In a separate multivariate Cox regression analysis, no visual or CALIPER CT variable retained significance against PVV after correction for age and gender (at a significance level of 0.01). Of the pulmonary functional indices, the only variable to maintain significance against PVV for mortality prediction after correction for age and gender was Kco. However, Kco remained a weaker predictor of mortality than PVV with identical *P* values to that shown in the multivariate analysis in Table 2. PVV remained the strongest single predictor of mortality in the CTD-ILD population.

### Automated stratification of CTD-ILD patients

The CTD-ILD cohort was stratified into three outcome groups using automated pairwise dissimilarity analyses. The disease extents of the various CT parenchymal patterns identified by CALIPER are pictorially represented for the three outcome groups as glyphs in Fig. 1. Demographic, CT and functional characteristics of the three groups are summarised in Table 1, whilst significant differences in CT and functional variables between automated stratified groups are shown in Additional file 1: Table S4.

Significant differences across all three groups were identified for FVC, DLco, TLC and CPI, and all CALIPER measures of fibrosis except honeycombing. Similarly, visual CT markers of fibrosis including fibrosis extent, reticular pattern and traction bronchiectasis were significantly different across all groups. CALIPER-derived PVV was also significantly different across all three groups.

### Evaluation of automated stratified groups against mortality

**Fig. 2:**
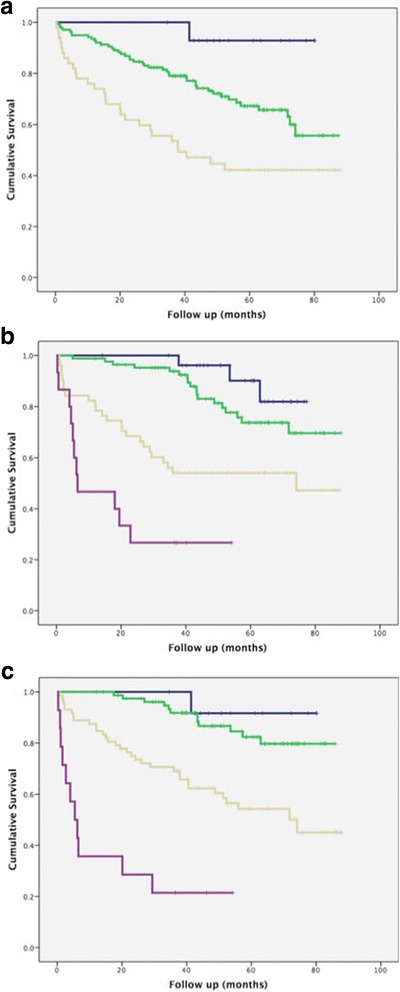
**a** Kaplan–Meier survival curve demonstrating differences in outcome for patients with connective tissue disease related-interstitial lung disease separated according to automated stratified groups. Group 1 (blue; mean survival 77.4 ± 2.7 months, n = 15), Group 2 (green; mean survival 66.4 ± 2.7 months, n = 138), Group 3 (yellow; mean survival 47.9 ± 5.2 months, n = 50). Log rank test *P* < 0.0001. **b** Kaplan–Meier survival curve demonstrating differences in outcome for patients with connective tissue disease related-interstitial lung disease separated according to the ILD-GAP model. Group 1 (blue; mean survival 73.3 ± 2.2 months, n = 28), Group 2 (green; mean survival 74.9 ± 2.7 months, n = 85), Group 3 (yellow; mean survival 53.6 ± 5.1 months, n = 51), Group 4 (magenta; mean survival 20.7 ± 5.5 months, n = 15). Log rank test *P* < 0.0001. **c** Kaplan–Meier survival curve demonstrating differences in outcome for patients with connective tissue disease related-interstitial lung disease separated according to the Stratified-CT model. Group 1 (blue; mean survival 77.0 ± 3.1 months, n = 13), Group 2 (green; mean survival 77.1 ± 2.3 months, n = 80), Group 3 (yellow; mean survival 57.5 ± 4.1 months, n = 72), Group 4 (magenta; mean survival 17.2 ± 5.6 months, n = 14). Log rank test *P* < 0.0001

Survival curves for the patients comprising the three automated stratified groups are shown in Fig. 2a (Log rank test *P* < 0.0001). Group 1 patients: n = 15; mean survival = 77.4 ± 2.7 months), group 2: n = 138; mean survival 66.4 ± 2.7 months, group 3: n = 50; mean survival 47.9 ± 5.2 months. The distribution of CTD-ILD diagnoses between groups is given in Table 3. Group 3 patients had the worst outcome and included all CTD diagnoses except SLE. Half of the patients with mixed connective tissue disease and almost a third of patients with RA, primary Sjögren’s syndrome, and polymyositis and dermatomyositis were included in the poor outcome group.

**Table 3:** Distribution of connective tissue disease-related interstitial lung disease diagnoses across the three groups derived using automated stratification with percentages in parentheses

	RA N = 50	SSc N = 65	SjS N = 10	MCTD N = 16	Myositis N = 23	Overlap N = 36	SLE N = 3
Group 1	4 (8)	5 (8)	0 (0)	2 (13)	1 (4)	3 (8)	0 (0)
Group 2	31 (62)	48 (74)	7 (70)	6 (38)	15 (65)	28 (78)	3 (100)
Group 3	15 (30)	12 (18)	3 (30)	8 (50)	7 (30)	5 (14)	0 (0)

*RA* rheumatoid arthritis, *SSc* systemic sclerosis, *SjS* Sjögrens syndrome, *MCTD* mixed connective tissue disease, *Overlap* overlap connective tissue disease, *SLE* systemic lupus erythematosus

On univariate Cox regression analysis, the automated stratified groups (n = 203) were strongly predictive of mortality (hazard ratio (HR) = 2.45, confidence interval (CI) 1.60–3.75, *P* < 0.0001). On bivariate mortality analyses, the automated stratified groups were stronger determinants of outcome than any single CT or pulmonary function index. In a bivariate mortality analysis with patient age, both variables were strongly independently predictive of mortality (age: HR = 1.07, CI 1.04–1.09, *P* < 0.0001; and automated stratified groups: HR = 2.98, CI 1.92–4.65, *P* < 0.0001).

### Comparison of automated stratified groups against patient outcome models

The ILD-GAP, Stratified CT, and Stratified CT-GAP models were each highly predictive of mortality on univariate analysis (Stratified CT: n = 203, HR = 3.18, CI 2.25–4.50, *P* < 0.0001; ILD-GAP: n = 179, HR = 2.89, CI 2.06–4.06, *P* < 0.0001; Stratified CT-GAP: n = 179, HR = 2.26, CI 1.76–2.91, *P* < 0.0001). Only 179 patients were evaluated in the ILD-GAP and Stratified CT-GAP models as 24 patients did not have FVC or DLco measurements. When the same 24 patients were excluded from the Stratified CT model, model strength improved (Stratified CT: n = 179, HR = 3.77, CI 2.51–5.66, *P* < 0.0001). In subsequent analyses, only the 179 patients common to the three models were compared.

When the Stratified CT and the ILD-GAP models were evaluated using bivariate Cox mortality analysis, the Stratified CT model was a stronger predictor of mortality (Stratified CT: n = 179, HR = 2.49, CI 1.54–4.01, *P* = 0.0002; ILD-GAP: n = 179, HR = 1.85, CI 1.24–2.76, *P* = 0.003). The results were maintained on bootstrapping of 1000 samples (Stratified CT: n = 179, *P* = 0.001, CI 0.41–1.52; ILD-GAP: n = 179, *P* = 0.003, CI 0.20–1.09).

Survival curves for the 179 CTD-ILD patients separated according to the ILD-GAP model and the same 179 CTD-ILD patients separated according to the Stratified CT model are demonstrated in Fig. 2b and c, respectively. The relatively reduced HR demonstrated for the Stratified-GAP model was a consequence of its wider 7-point scale, but the narrow confidence interval range highlights its strength over the other models.

**Fig. 3:**
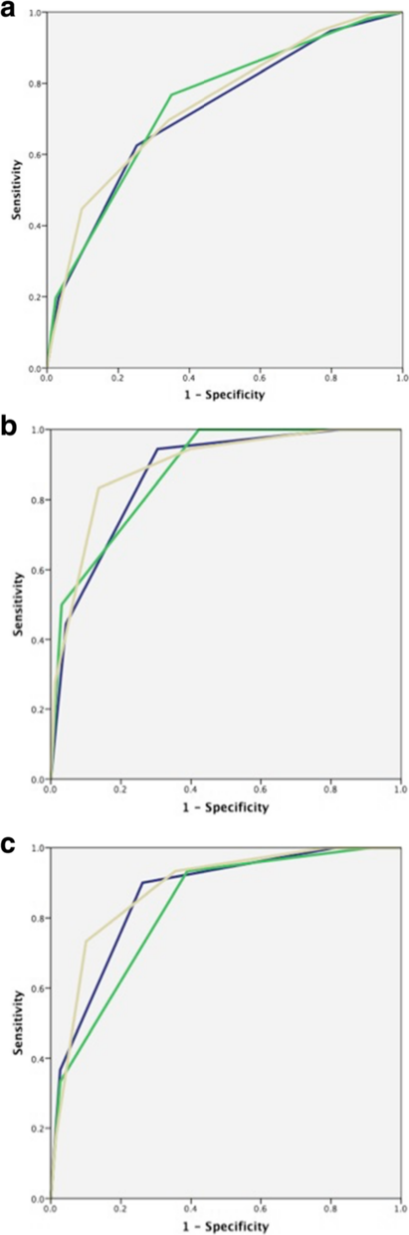
**a** Receiver operating characteristic (ROC) curves demonstrating sensitivity and specificity for overall mortality prediction using three models: ILD-GAP model (Blue; AUROCC = 0.72, *P* < 0.0001, CI 0.64–0.80), Stratified-CT Model (Green; AUROCC = 0.74, *P* < 0.0001, CI 0.66–0.82), Stratified CT-GAP model (Yellow; AUROCC = 0.74, *P* < 0.0001, CI 0.66–0.82). **b** ROC curves demonstrating sensitivity and specificity for prediction of death within a year from the patients initial CT scan using three models: ILD-GAP model (Blue; AUROCC = 0.87, *P* < 0.0001, CI 0.80–0.95), Stratified-CT Model (Green; AUROCC = 0.88, *P* < 0.0001, CI 0.81–0.95), Stratified CT-GAP model (Yellow; AUROCC = 0.89, *P* < 0.0001, CI 0.82–0.97). **c** ROC curves demonstrating sensitivity and specificity for prediction of death within 2 years from the patients initial CT scan using three models: ILD-GAP model (Blue; AUROCC = 0.86, *P* < 0.0001, CI 0.80–0.93), Stratified-CT Model (Green; AUROCC = 0.83, *P* < 0.0001, CI 0.75–0.90), and Stratified CT-GAP model (Yellow; AUROCC = 0.88, *P* < 0.0001, CI 0.82–0.95)

On ROC curve analysis, prediction of mortality at 1 year, 2 years and overall mortality was analysed for the three models: ILD-GAP model, Stratified CT model, and the Stratified CT-GAP model (Fig. 3); 18/179 patients died within a year of the CT scan being performed, whilst 30/179 patients died within 2 years. The area under the ROC curve (AUROCC) was consistently higher for the Stratified CT-GAP model when compared to the ILD-GAP model, and was higher for 1-year and overall mortality with the Stratified CT model over the ILD-GAP model.

## Discussion

Our study has demonstrated for the first time, that, across the range of CTD-ILD diagnoses, a computer-derived CT parameter, the pulmonary vessel volume, is an independent predictor of mortality. Furthermore, the PVV is a stronger predictor of mortality than all other CT and pulmonary function variables following correction for age and gender. In addition, automated stratification of CALIPER-derived CT variables identifies patient groups with distinct characteristics, and three automated stratified groups demonstrated significantly different functional profiles and patient outcomes. When the functional indices (FVC, DLco) in the ILD-GAP model were substituted with the automated stratified groups, the new Stratified CT model improved mortality prediction when compared to the ILD-GAP model. When the automated stratified groups were subsequently combined with the ILD-GAP model (Stratified CT-GAP model), mortality prediction was further augmented. Accordingly, automated stratified CALIPER CT variables have the potential to be used as an alternative to, or combined with, functional indices to predict outcome in CTD-ILD patients.

Our observations are particularly relevant given a recent editorial which articulated the need to improve the identification of distinct disease phenotypes in patients with rheumatoid arthritis related-ILD to aid risk prediction and diagnosis [30]. Apart from systemic sclerosis, most studies in the CTD-ILDs have been constrained by small patient numbers. Accordingly, there is a growing need to combine patient cohorts across centres to generate more substantial and inclusive datasets [30]. Although CT evaluation is near ubiquitous in the setting of known or suspected CTD-ILD, the complexities and inconsistencies associated with visual CT scoring demand more robust alternatives for the quantification of disease patterns and extents.

Computer analysis of CTs in CTD-ILD populations is an attractive alternative to visual scoring and when combined with the unbiased nature of automated stratification, may allow the identification of patient phenotypes that are visually subliminal. In addition, the strength of DLco as a predictor of outcome in CTD-ILD may well be diminished in multicentre cohorts given the variation associated with DLco measurements across laboratories [31], further emphasising CT evaluation as a potential outcome measure in patients with CTD-ILD. In this regard, the ability of automated stratification to substitute for DLco and FVC measured at a single institution, without loss of strength in outcome prediction, argues for consideration of computer-based CT analysis in future multicentre CTD-ILD studies.

The improved strength of the Stratified CT-GAP model over the ILD-GAP model identified in the current study is largely a consequence of a confounding effect of the normal range of pulmonary function when PFTs are stratified as thresholds. The range of normal pulmonary function values extends across the range of 80–120% of predicted values based on patient age, gender, race and height. As a result, in a staging system, if a patient lies close to a lung function threshold, small differences in predicted normal values will have a major impact on how the patient is staged, shifting them above and below thresholds. For example, if a patient started with a predicted FVC at 120% and lost 35% of predicted lung function they would remain as GAP stage 1. However, if the patient started at a predicted FVC of 80% and lost 35% of predicted FVC, they would fall into GAP stage 3. Consequently, the normal range has a dramatic effect on the severe end of the spectrum of disease in determining where someone lies on the GAP scale.

A similar limitation of a “normal range” is not present in morphological CT variables however and CT variables can therefore serve to modify confounding effects associated with clustering around PFT thresholds as identified in a previous study evaluating a scleroderma staging system [6]. Goh et al. [6] showed that threshold measures of CT (Hazard ratio [HR] = 2.5) and PFT (HR = 2.1 for an FVC threshold) variables were significantly weaker when analysed alone, but improved considerably when structure and function were combined (HR = 3.5). Similarly, in the current study, the ILD-GAP model was a less sensitive predictor of mortality secondary to the clustering of individuals around PFT thresholds, an effect that was partially ameliorated following amalgamation of the Stratified CT score to the ILD-GAP model.

The current study is the first of its kind to evaluate mortality prediction in CTD patients using computer-based volumetric CT analysis. Several previous studies in CTD patients analysing CT scans with computer algorithms have utilized interspaced high-resolution CT imaging [11, 12], precluding the robust evaluation and differentiation of patterns such as honeycombing and emphysema. The remaining computer-based studies have evaluated the lung according to its simple density characteristics, deriving metrics of histogram skewness and kurtosis [32–34]. Such metrics have been shown to correlate poorly with other markers of disease severity and with mortality in IPF [15] and are relatively unsophisticated compared to modern structural and textural analytic techniques [27]. Furthermore, only the studies by Marten et al. [32, 33] evaluated computer scores against physiological indices whilst the remaining studies compared computer-based scores with visual CT scoring. No studies to date have evaluated computer scores against mortality in patients with CTD.

CALIPER has advantages over most quantitative tools by virtue of its volumetric structural and textural analysis of the lung, which, for example, enables low attenuation areas of the lung to be distinguished as representing either honeycombing or emphysema [27]. Similarly, volumetric analysis allows quantitation of features that cannot be resolved visually, such as the percentage of the lung volume composed of vessels [25]. A glyph distils CALIPERs quantitative data into a format that is easily deciphered by the non-specialist in a busy clinic setting, which may have crossover utility for both rheumatologists and pulmonologists in the evaluation of patients with CTD-ILD. Whilst the glyph presentations are a by-product of CALIPER analysis we do not wish to give undue prominence to them in the current study however, since it is based on population characteristics rather than individual patient/glyph appearances. Interrogating an individual glyph, which simplifies complex spatial patterns of disease morphology and extent, is an inferior exercise when compared to the modelling analyses conducted in the current study. To derive absolute conclusions about an individual’s likely outcome based solely on a glyph would be misleading.

There are very few large-scale studies that have evaluated the ability of CT variables to predict mortality across all CTD subtypes. A study by Walsh et al. [7] evaluated CTs and pulmonary function indices in 168 patients with various CTDs and found that traction bronchiectasis severity and honeycombing extent scored visually along with DLco were independently predictive of mortality. In the present study, across all CTD-ILD patients, when visually scored CT parameters were analysed alone, visual honeycombing and traction bronchiectasis severity scores were also independently predictive of mortality. However, when combined with CALIPER CT variables and PFTs, however, visual honeycombing and traction bronchiectasis scores did not retain prognostic significance.

The association between pulmonary hypertension and connective tissue diseases has long been recognised [35], and supervening pulmonary hypertension is associated with a poor outcome across the range of CTDs [36–38]. It would therefore be logical to assume that the mortality signal associated with PVV reflected a new imaging marker of pulmonary hypertension. However, as with our observations in patients with idiopathic pulmonary fibrosis [25, 39], we identified only weak linkages between PVV and both RVSP and Kco. Indeed, Kco and PVV were independently predictive of mortality across the range of CTD patients. The findings suggest that the PVV signal does not primarily reflect the severity of pulmonary hypertension, or indeed act as a key marker of damage to the vascular compartment of the lung. The counter-intuitive relationship between PVV and the extent of ILD identified on CT may, as previously postulated, be explained by local increased vascular pressures within fibrotic regions of the lung that result in blood diversion to spared lung regions. As fibrosis worsens and vessel size and number (above a size threshold recognised by CALIPER) increase in non-fibrotic regions of the lung, the accompanying increase in CALIPER PVV may effectively act as a surrogate marker of ILD extent [39].

The superiority of PVV in predicting mortality over CALIPER and visually scored total ILD extents may relate to the specific pathophysiologic changes that develop in the lung secondary to fibrosis. As fibrosis worsens, the lung contracts with the result that the extent of fibrosis, when measured volumetrically or expressed as a proportion of the total lung volume may, in fact, decrease. Consequently, in a patient with more severe disease, a volumetric CT score of fibrosis extent underestimates fibrosis severity. PVV avoids such a pitfall, as it is a parameter that increases in line with fibrosis extent. Evaluation of PVV as a prognostic marker in the fibrosing lung diseases remains in its infancy; however, results from the current study argue for further detailed study of the variable in other fibrosing lung diseases as well as evaluation of PVV as a marker of deterioration on serial CT evaluation.

There are some limitations to this retrospective study. Firstly, the individual CTD-ILD diagnoses making up the cohort were not evenly distributed, for example, there were large numbers of RA-ILD and systemic sclerosis-ILD patients but very few cases of SLE. In mitigation, however, given that the study population represented a consecutive cohort of new clinic presentations, the case mix arguably represents a real-world caseload. Secondly, there were only 14 patients in automated stratified group 1, limiting the strength of statistical relationships between groups. A consequence was that the remaining patients were split into two groups generating dichotomous good and bad outcome groups. Since most management decisions are binary with regard to giving or withholding medication, a two-group model is usually preferable to a multiple group model where managing patients in intermediate outcome groups is problematic. It could also be argued that some patients with an apparently good outcome may turn out to have a delayed poor outcome once treatment benefits have dissipated. Such a reservation is common to a great many studies, and yet primarily evaluating all patients at presentation does, at least, provide a satisfactory spread of disease severity, including some patients with earlier disease and others with more advanced disease. There are limitations associated with making exact prognostic separations based on baseline evaluation and conclusions reached at a single point in time should retain some flexibility to enable modification by observed changes in subsequent disease behaviour. Finally, an external validation cohort would ideally have been used to confirm our findings; however, the scarcity of large, well characterised fibrosing lung disease cohorts remains a recognised constraint.

## Conclusions

In conclusion, we have demonstrated that, in a large mixed population of CTD-ILD patients, CALIPER pulmonary vessel volume was the CT variable that best predicted mortality and may be a new prognostic index. When automated stratified CALIPER variables were substituted for the functional indices in the ILD-GAP index, mortality prediction was strengthened. Computer analysis and automated stratification of CTs may therefore represent a viable alternative to visual CT scoring and evaluation of functional indices in patients with CTD-ILD, and demonstrates added value when combined with outcome prediction models such as the ILD-GAP model.

## Additional file

Additional file 1
**Table S1.** Lobar visual scores were adjusted using scintigraphic and gas dilution measures of the physiological contribution of each lobe to the total lung volume in health (top row). The figure was divided by the proportion of each lung representing a lobe (16.7%), or in the case of the left upper lobe, which included the lingula, two lobes (33.3%). **Table S2.** Single determination standard deviation values of visual CT scores for connective tissue disease-related interstitial lung disease cases. **Table S3.** Patient age, gender, smoking status and measures of pulmonary function indices, CALIPER and visually scored CT parameters and echocardiography data for the four groups of the ILD-GAP index. Data represent mean values with standard deviations. CTD, connective tissue disease; FEV1, forced expiratory volume in one second; FVC, forced vital capacity; DLco, diffusing capacity for carbon monoxide; Kco, carbon monoxide transfer coefficient; TLC, total lung capacity; CPI, composite physiologic index; ILD, interstitial lung disease; GGO, ground glass opacity; PVV, pulmonary vessel volume; TxBx, traction bronchiectasis; PA, pulmonary artery; AAo, ascending aorta; RVSP, right ventricular systolic pressure. **Table S4.** *P* values demonstrating differences between automated stratified groups calculated using one-way ANOVA with Bonferroni correction for continuous variables and t-test with Bonferroni correction for categorical variables. ILD, interstitial lung disease; PA, pulmonary artery; Ao, ascending aorta; HC, honeycombing; DLco, diffusing capacity for carbon monoxide; Kco, carbon monoxide transfer coefficient; CPI, composite physiologic index; RVSP, right ventricular systolic pressure. * not significant. **Figure S1.** CONSORT diagram illustrating the selection of patients for the final study population. ILD, interstitial lung disease; CTD, connective tissue disease; IPAF, interstitial pneumonia with autoimmune features; LCH, Langerhans cell histiocytosis; LAM, lymphangioleiomyomatosis; CT, computed tomography. (DOCX 67 kb)

## Abbreviations

ILD: interstitial lung disease
CT: computed tomography
PVV: pulmonary vessel volume
FEV1: forced expiratory volume in one second
FVC: forced vital capacity
DLco: diffusing capacity for carbon monoxide
Kco: carbon monoxide transfer coefficient
CPI: composite physiologic index
TLC: total lung capacity
ILD-GAP: interstitial lung disease gender, age, physiology model
CTD-ILD: connective tissue disease related interstitial lung disease
CALIPER: Computer-Aided Lung Informatics for Pathology Evaluation and Rating
PFT: pulmonary function test
HR: hazard ratio
CI: confidence interval
IPF: idiopathic pulmonary fibrosis
ANOVA: analysis of variance
ROC: receiver operating characteristic
RA: rheumatoid arthritis
SLE: systemic lupus erythematosus
RVSP: right ventricular systolic pressure
