# Supplementary material for: Evaluation of computer-based computer tomography stratification against outcome models in connective tissue disease-related interstitial lung disease: a patient outcome study
Source: BMC Med. 2016 Nov 23;14:190. doi: 10.1186/s12916-016-0739-7 (PMC5120564; doi:10.1186/s12916-016-0739-7)
Supplement: Additional file 1 — Table S1. Lobar visual scores were adjusted using scintigraphic and gas dilution measures of the physiological contribution of each lobe to the total lung volume in health (top row). The figure was divided by the proportion of each lung representing a lobe (16.7%), or in the case of the left upper lobe, which included the lingula, two lobes (33.3%). Table S2. Single determination standard deviation values of visual CT scores for connective tissue disease-related interstitial lung disease cases. Table S3. Patient age, gender, smoking status and measures of pulmonary function indices, CALIPER and visually scored CT parameters and echocardiography data for the four groups of the ILD-GAP index. Data represent mean values with standard deviations. CTD, connective tissue disease; FEV1, forced expiratory volume in one second; FVC, forced vital capacity; DLco, diffusing capacity for carbon monoxide; Kco, carbon monoxide transfer coefficient; TLC, total lung capacity; CPI, composite physiologic index; ILD, interstitial lung disease; GGO, ground glass opacity; PVV, pulmonary vessel volume; TxBx, traction bronchiectasis; PA, pulmonary artery; AAo, ascending aorta; RVSP, right ventricular systolic pressure. Table S4. P values demonstrating differences between automated stratified groups calculated using one-way ANOVA with Bonferroni correction for continuous variables and t-test with Bonferroni correction for categorical variables. ILD, interstitial lung disease; PA, pulmonary artery; Ao, ascending aorta; HC, honeycombing; DLco, diffusing capacity for carbon monoxide; Kco, carbon monoxide transfer coefficient; CPI, composite physiologic index; RVSP, right ventricular systolic pressure. * not significant. Figure S1. CONSORT diagram illustrating the selection of patients for the final study population. ILD, interstitial lung disease; CTD, connective tissue disease; IPAF, interstitial pneumonia with autoimmune features; LCH, Langerhans cell histiocytosis; LAM, lymphangioleiomyomat [file 12916_2016_739_MOESM1_ESM.docx]

**Additional file 1: APPENDICES**

**CT Protocols**

The CT scans were obtained using a 64-slice multiple detector CT scanner (Somatom Sensation 64, Siemens, Erlangen, Germany) or a 4-slice multiple detector CT scanner (Siemens Volume Zoom, Siemens, Erlangen, Germany. To satisfy requirements for processing by the CALIPER algorithm, all scans were reconstructed using a high spatial frequency, B70 kernel (Siemens, Munich, Germany). All patients were scanned from lung apices to bases, supine, at full inspiration, with 1·0mm section thicknesses using a peak voltage of 120kVp with tube current modulation (range 30-140 mA). Images were viewed at window settings optimized for the assessment of the lung parenchyma (width 1500 H.U.; level -500 H.U.).

**Pulmonary function tests**

Pulmonary function tests were analyzed if performed within 3 months of the corresponding CT scan according to established protocols [^1^](#_ENREF_1). Spirometry (Jaeger Master screen PFT, Carefusion Ltd., Warwick, UK), plethysmographic lung volumes (Jaeger Master screen Body, Carefusion Ltd., Warwick, UK), and diffusion capacity for carbon monoxide (DLco) (Jaeger Master screen PFT, Carefusion Ltd., Warwick. UK) Parameters assessed: forced expiratory volume in one second (FEV1), forced vital capacity (FVC), total lung capacity (TLC), transfer coefficient of the lung for carbon monoxide (Kco) and single breath carbon monoxide diffusing capacity corrected for hemoglobin concentration (DLco). The composite physiologic index (CPI) was calculated using the formula: 91·0 - (0·65 x % predicted DLco) - (0·53 x % predicted FVC) + (0·34 x % predicted FEV1).

**Echocardiography**

A measure of pulmonary hypertension was obtained from right ventricular systolic pressure (RVSP) evaluated on transthoracic echocardiography, and only included if performed within 3 months of the CT scan.

**Adjustment for visual lobar scores using normalized lobar volumes**

As fibrosis develops, and in the case of IPF, shrinks the lower lobes disproportionately, the true extent of lung fibrosis can be underestimated when using volumetric scoring systems. The shrunken contracted lower lobes contribute relatively smaller volumes toward the total lung volume, than would be the case in health. As a consequence, lower lobes that might have been 25% of the lung volume in health, when shrunken to 10% of the total lung volume secondary to fibrosis, cannot reflect the true extent of disease within the lungs when using a volumetric score as was the case with CALIPER, and traditional visual scoring of 5 axial CT sections.

A lobar score, as used in the visual scoring of CT scans in the current study however, more accurately relates the extent of fibrosis within the lung as each lobe is categorized as a discrete unit, regardless of its post-fibrosis induced volume loss. A potential inaccuracy with visual lobar scoring lies in the relatively high proportional weighting given to the middle lobes, which are each quantified as one sixth of the lung. To account for this, the lobar scores of each individual visual scorer were adjusted to reflect the physiological contribution of each lobe to the total lung volume in health (Additional file 1: Table S1), as characterized using scintigraphic and gas dilution techniques.[^2^](#_ENREF_2) Therefore the right upper lobe scores were multiplied by 16.4/16.7, where 16.4% represented the physiological volume of the right upper lobe in health and 16.7% represented a sixth of the lung volume. The left upper and middle lobes were considered together and adjusted accordingly.

|  | **Right upper lobe** | **Left upper lobe** | **Right middle lobe** | | **Right lower lobe** | | **Left lower lobe** |
| --- | --- | --- | --- | --- | --- | --- | --- |
| **Physiological volumes of lobes in health (% of total lung volume)** | 16.4 | 26.5 | 9.6 | 26.4 | | 24.0 | |
| **Standardized contribution of a lobe to total lung volume (%)** | 16.7 | 33.3 | 16.7 | 16.7 | | 16.7 | |

Table S1. Lobar visual scores were adjusted using scintigraphic and gas dilution measures of the physiological contribution of each lobe to the total lung volume in health (top row). The figure was divided by the proportion of each lung representing a lobe (16.7%), or in the case of the left upper lobe, which included the lingula, two lobes (33.3%).

**Consensus formulation for visual scores**

The identification of systematic biases in visual scores was achieved by plotting the spread of differences in parenchymal pattern scores between observers. The most disparate 5% (two standard deviations) of values were arbitrated by a third scorer for all parameters thereby minimizing bias within the original scorers. If a single parenchymal subtype extent was changed at consensus, the other parameters were modified, following CT review, to retain an overall sum of 100% for the four parenchymal subtypes. Similarly, if the lobar percentages of total interstitial disease or emphysema varied, the other parameter extent was rescored.

**CALIPER CT evaluation**

Data processing: Initial data processing steps involved extraction of the lung from the surrounding thoracic structures and segmentation into upper, middle and lower zones. Lung segmentation was performed with an adaptive density-based morphological approach [^3^](#_ENREF_3) whilst airway segmentation involved iterative three-dimensional region growing, density thresholding (thresholds including -950HU and -960HU) and connected components analysis. Pulmonary vessels were extracted using optimized multi-scale tubular structure enhancement filters [^4^](#_ENREF_4). Parenchymal tissue type classification was applied to 15x15x15 voxel volume units using texture analysis, computer vision-based image understanding of volumetric histogram signature mapping features and 3D morphology [^5^](#_ENREF_5). The CALIPER tool was trained by sub-specialty thoracic radiologist consensus assessment of pathologically confirmed datasets [^5^](#_ENREF_5), [^6^](#_ENREF_6).

The mathematical equation to compare a pair of patients based on the distribution of eight patterns (Normal lung, ground glass opacity, honeycombing, reticular pattern, pulmonary vessels, and mild LAA, moderate LAA and severe LAA) across the six zones (LU, LM, LL, RU, RM, RL) is given by,

$$D(A,B)=\sum_{i=1:8} \frac{|{}_{abn}A_{i}^{L}-{}_{abn}B_{i}^{L}|}{\max({}_{abn}A_{i}^{L},{}_{abn}B_{i}^{L})}+\sum_{j=1:6} (\frac{{}_{vol}A^{R_{j}}}{{}_{vol}A^{L}}\times(1+\frac{|{}_{vol}A_{i}^{R_{j}}-{}_{vol}B_{i}^{R_{j}}|}{\max({}_{vol}A_{i}^{R_{j}},{}_{vol}B_{i}^{R_{j}})})\times\sum_{k=1:8} \frac{|{}_{abn}A_{k}^{R_{j}}-{}_{abn}B_{k}^{R_{j}}|}{\max({}_{abn}A_{k}^{R_{j}},{}_{abn}B_{k}^{R_{j}})})$$

where, ${}_{abn}X_{i}^{L(or)R_{j}}$represents the percentage abnormality distribution of *i^th^* (of eight) CT pattern in the whole lung (L) (or) in *j^th^* (of six) region of the lung (*R_j_*). ${}_{vol}X^{L(or)R_{j}}$represents the total volume of the lung (L) (or) in *j^th^* region of the lung (*R_j_*). *X* represents a patient’s CT lung volume.

| **Visual CT Variable (n = 207)** |  | **Single determination standard deviation** |
| --- | --- | --- |
| CT Interstitial lung disease extent |  | 11.28 |
| CT Ground glass density |  | 15.97 |
| CT Reticular pattern |  | 8.84 |
| CT Honeycombing |  | 5.26 |
| CT Consolidation |  | 1.51 |
| CT Total emphysema |  | 5.20 |
| CT Mosaic attenuation |  | 3.43 |
| CT Traction bronchiectasis severity |  | 1.36 |

Table S2. Single determination standard deviation values of visual CT scores for connective tissue disease-related interstitial lung disease cases.

| Variable | ILD-GAP  Group 1 | ILD-GAP  Group 2 | ILD-GAP  Group 3 | ILD-GAP  Group 4 |
| --- | --- | --- | --- | --- |
| Units are percentage unless stated | (n=28 unless stated) | (n=85 unless stated) | (n=51 unless stated) | (n=15 unless stated) |
| Median Age (years) | 54.5 | 56 | 58.5 | 71 |
| Male/female | 3/25 | 18/67 | 28/23 | 8/7 |
| Survival (alive/dead) | 25/3 | 67/18 | 27/24 | 4/11 |
| Never/ex-smokers | 18/9 (27) | 56/27 | 26/25 | 7/8 |
| Follow up time (months) | 55.8 ± 15.0 | 52.7 ± 19.6 | 40.1 ± 27.8 | 17.4 ± 17.2 |
| FEV1 % predicted | 84.4 ± 14.9 (28) | 72.0 ± 15.5 (85) | 59.9 ± 18.5 (51) | 54.0 ± 9.7 (15) |
| FVC % predicted | 92.1 ± 15.0 (28) | 75.0 ± 17.6 (85) | 59.3 ± 18.1 (51) | 50.0 ± 10.6 (15) |
| DLco % predicted | 58.0 ± 10.0 (28) | 41.7 ± 11.4 (85) | 30.0 ± 10.1 (51) | 24.7 ± 7.2 (15) |
| Kco % predicted | 77.1 ± 14.0 (28) | 67.6 ± 16.3 (85) | 59.9 ± 18.4 (51) | 58.3 ± 17.9 (15) |
| TLC% predicted | 89.1 ± 11.7 (27) | 73.5 ± 14.8 (76) | 60.3 ± 11.3 (48) | 54.0 ± 11.6 (14) |
| CPI | 33.2 ± 8.3 (28) | 48.6 ± 10.4 (85) | 60.4 ± 9.1 (51) | 66.8 ± 6.9 (15) |
| CALIPER ILD extent | 7.0 ± 7.7 | 18.0 ± 16.8 | 29.6 ± 20.3 | 27.8 ± 18.0 |
| CALIPER Fibrosis extent | 1.9 ± 1.7 | 4.2 ± 3.7 | 8.1 ± 5.9 | 10.8 ± 7.0 |
| CALIPER GGO | 5.1 ± 6.9 | 13.9 ± 15.6 | 21.5 ± 17.9 | 17.1 ± 14.3 |
| CALIPER Reticular pattern | 1.8 ± 1.7 | 3.7 ± 3.4 | 7.1 ± 5.0 | 9.7 ± 6.5 |
| CALIPER Honeycombing | 0.1 ± 0.1 | 0.4 ± 1.1 | 1.0 ± 2.5 | 1.0 ± 1.5 |
| CALIPER Emphysema | 0.7 ± 2.5 | 0.5 ± 2.5 | 0.3 ± 1.0 | 0.4 ± 0.8 |
| CALIPER PVV | 2.9 ± 1.0 | 3.8 ± 1.1 | 5.3 ± 1.9 | 5.4 ± 2.1 |
| CALIPER Normal lung | 89.3 ± 8.8 | 77.7 ± 1.5 | 64.8 ± 21.9 | 66.3 ± 19.3 |
| Visual ILD extent | 36.0 ± 21.7 | 45.7 ± 23.8 | 56.7 ± 21.6 | 62.6 ± 23.0 |
| Visual fibrosis extent | 12.7 ± 9.8 | 23.8 ± 14.0 | 39.4 ± 18.8 | 38.9 ± 23.2 |
| Visual GGO | 22.5 ± 21.0 | 21.3 ± 21.7 | 16.2 ± 15.7 | 23.0 ± 19.6 |
| Visual Reticular pattern | 12.4 ± 9.7 | 21.7 ± 13.8 | 33.1 ± 16.8 | 34.7 ± 20.3 |
| Visual Honeycombing | 0.2 ± 0.7 | 2.2 ± 4.9 | 6.3 ± 11.9 | 4.2 ± 9.1 |
| Visual Consolidation | 0.7 ± 2.6 | 0.3 ± 1.2 | 0.8 ± 2.4 | 0.4 ± 0.9 |
| Visual Emphysema | 3.9 ± 12.1 | 2.6 ± 7.1 | 5.3 ± 12.4 | 3.2 ± 6.7 |
| Visual Mosaicism | 2.0 ± 8.0 | 1.7 ± 4.6 | 1.1 ± 2.2 | 2.4 ± 4.3 |
| Visual TxBx (max score 18) | 2.8 ± 2.5 | 5.0 ± 3.3 | 7.0 ± 3.1 | 8.5 ± 5.6 |
| Main PA diameter (mm) | 27.3 ± 3.2 | 30.6 ± 4.3 | 32.9 ± 4.9 | 32.4 ± 4.2 |
| AAo diameter (mm) | 30.4 ± 3.3 | 31.6 ± 3.8 | 33.0 ± 3.9 | 33.1 ± 3.7 |
| RVSP (mmHg) | 29.5 ± 6.5 (11) | 35.8 ± 14.2 (43) | 43.9 ± 18.2 (26) | 46.6 ± 22.3 (9) |

Table S3. Patient age, gender, smoking status and measures of pulmonary function indices, CALIPER and visually scored CT parameters and echocardiography data for the four groups of the ILD-GAP index. Data represent mean values with standard deviations. CTD=connective tissue disease, FEV1 = forced expiratory volume in one second, FVC = forced vital capacity, DLco = diffusing capacity for carbon monoxide, Kco=carbon monoxide transfer coefficient, TLC=total lung capacity, CPI=composite physiologic index, ILD=interstitial lung disease, GGO=ground glass opacity, PVV = pulmonary vessel volume, TxBx = traction bronchiectasis, PA=pulmonary artery, AAo=ascending aorta, RVSP=right ventricular systolic pressure.

| Variable  (Units are percentage unless stated) | Stratified Group  1 vs 2 | Stratified Group  2 vs 3 | Stratified Group  1 vs 3 |
| --- | --- | --- | --- |
|  | (P values) | (P values) | (P values) |
| Median Age (years) | 1.00* | 1.00* | 1.00* |
| Male/female | 0.31* | 1.00* | 0.76* |
| Survival (alive/dead) | 0.15* | 0.006 | 0.003 |
| Never/ex-smokers | 1.00* | 1.00* | 1.00* |
| FEV1 % predicted | 0.30* | <0.0001 | 0.0003 |
| FVC % predicted | 0.03 | <0.0001 | <0.0001 |
| DLco % predicted | 0.001 | <0.0001 | <0.0001 |
| Kco % predicted | 1.00* | 0.007 | 0.04 |
| TLC% predicted | 0.0004 | <0.0001 | <0.0001 |
| CPI | 0.0004 | <0.0001 | <0.0001 |
| CALIPER ILD extent | 0.001 | <0.0001 | <0.0001 |
| CALIPER Fibrosis extent | 0.009 | <0.0001 | <0.0001 |
| CALIPER GGO | 0.04 | <0.0001 | <0.0001 |
| CALIPER Reticular pattern | 0.007 | <0.0001 | <0.0001 |
| CALIPER Honeycombing | 1.00* | 0.003 | 0.04 |
| CALIPER Emphysema | 0.82* | 0.43* | 1.00* |
| CALIPER PVV | 0.001 | <0.0001 | <0.0001 |
| Visual ILD extent | 1.00* | <0.0001 | <0.0001 |
| Visual fibrosis extent | <0.0001 | <0.0001 | <0.0001 |
| Visual GGO | 0.03 | 0.78* | 0.24* |
| Visual Reticular pattern | 0.0002 | <0.0001 | <0.0001 |
| Visual Honeycombing | 0.91* | <0.0001 | 0.001 |
| Visual Emphysema | 1.00* | 1.00* | 1.00* |
| Visual TxBx (max score 18) | <0.0001 | <0.0001 | <0.0001 |
| RVSP (mmHg) | 1.00* | 0.03 | 0.02 |

Table S4. P values demonstrating differences between automated stratified groups calculated using one-way ANOVA with Bonferroni correction for continuous variables and T-test with Bonferroni correction for categorical variables. ILD=interstitial lung disease, PA=pulmonary artery, Ao=ascending aorta, HC=honeycombing, DLco = diffusing capacity for carbon monoxide, Kco=carbon monoxide transfer coefficient, CPI=composite physiologic index, RVSP=right ventricular systolic pressure. *=not significant.

1524 New ILD attendances

115 Sarcoid

337 Non-ILD diagnoses

- 46 Non ILD CTD
- 86 Infection/airways disease/cancer
- 26 Non fibrotic vasculitis
- 54 LCH, LAM, haemosiderosis
- 129 Other non-ILD diagnosis

1068 Patients in database

71 patients with IPAF diagnosis excluded

718 Patients with non-CTD-ILD diagnosis

279 Patients with CTD-ILD diagnosis

74 Excluded - No departmental volumetric CT

203 Patients with CTD-ILD

(2 CT scans not compatible with CALIPER)

Figure S1. CONSORT diagram illustrating the selection of patients for the final study population. ILD = interstitial lung disease, CTD = connective tissue disease, IPAF = interstitial pneumonia with autoimmune features, LCH = Langerhans cell histiocytosis, LAM = lymphangioleiomyomatosis, CT = computed tomography.

**REFERENCES**

1. Quanjer PH. Standardized lung function testing. *Eur Respir J - Suppl*. 1993;**6**:1-100.

2. Pierce RJ, Brown DJ, Denison DM. Radiographic, scintigraphic, and gas-dilution estimates of individual lung and lobar volumes in man. *Thorax*. 1980;**35**(10):773-80.

3. Hu S, Hoffman EA, Reinhardt JM. Automatic lung segmentation for accurate quantitation of volumetric X-ray CT images. *IEEE Trans Med Imaging*. 2001;**20**(6):490-8.

4. Shikata H, McLennan G, Hoffman EA, et al. Segmentation of pulmonary vascular trees from thoracic 3D CT images. *Int J Biomed Imaging*. 2009:11.

5. Bartholmai BJ, Raghunath S, Karwoski RA, et al. Quantitative CT imaging of interstitial lung diseases. *J Thorac Imaging*. 2013;**28**(5):298-307.

6. Maldonado F, Moua T, Rajagopalan S, et al. Automated quantification of radiological patterns predicts survival in idiopathic pulmonary fibrosis. *Eur Respir J*. 2014;**43**(1):204-12.
